# Long-term physical and psychological outcome following traumatic hemipelvectomy

**DOI:** 10.1007/s00068-022-02151-2

**Published:** 2022-11-09

**Authors:** Galland Patrick, Krettek Christian, Ernst Jennifer, Sehmisch Stephan, Decker Sebastian

**Affiliations:** Trauma Department, Hannover Medical School, Carl-Neuberg-Str. 1, 30916 Hannover, Germany

**Keywords:** Traumatic hemipelvectomy, Amputation, Pelvic ring fracture, Quality of life

## Abstract

**Purpose:**

First time examination of the physical and psychological long-term outcome following traumatic hemipelvectomy.

**Methodology:**

In this study, all patients suffering from traumatic hemipelvectomy that were treated in a level-A trauma center since 1988 were retrospectively evaluated. The authors aimed to compare the physical and psychological outcome following primary amputation (A) vs. limb-preservation (LP) procedures. The patients were examined with a focus on pain, function, mobility and general health. As part of this examination, various scores were recorded, i.e., Majeed Score, Time up & Go or SF-36.

**Results:**

The following work showed 13 patients who had suffered a traumatic hemipelvectomy, 8 of whom survived. Five of these were available for subsequent clinical re-examination; of these, three patients underwent an amputation, while limb preservation was performed on two patients. Mean follow-up of the amputee group was after 12 years compared to 6.5 years following limb preservation. After limb preservation, both patients reported phantom limb pain at the affected leg, despite pain medication. The general state of health was assessed as 82/100 (A) and 45/100 (LP). The Majeed score was 61 (A) vs. 45 (LP). In the clinical examination, three out of five patients (2 LP, 1 A) showed peroneal palsy (PP). In the quality-of-life analysis based on the SF12/36 and the NHP, amputees scored higher than the patients who underwent limb preservation surgery.

**Conclusion:**

In our small patient cohort, satisfaction, pain and mobility tend to be better following primary amputation compared to limb preserving surgery.

## Introduction

Traumatic hemipelvectomy (THP) is a rare injury to the pelvis, accounting for only 0.6% of all pelvic fractures [9, 32, 34, 42]. The first case of traumatic hemipelvectomy was reported by Turnbull in 1978 [4, 6, 21, 30]. This specific injury is defined as the combination of an anterior pelvic ring fracture/transsymphyseal disruption and an iliosacral fracture on one or both sides, with avulsion of the external iliac vessels and severe stretch injury or disruption of the femoral and sciatic nerves [17, 32, 34, 42]. There were a few authors who subdivided this injury as complete or partial THP dependent on if the lower extremity was still attached to the trunk or not [16, 43]. In this study, both complete and partial THP were included. The most common accidental mechanisms causing traumatic hemipelvectomy include trauma from car and motorcycle crashes, as well as falls from great heights [6, 10]. Most patients with THP sustain multiple injuries [26, 28, 32]. The fatality rate of THP is approximately 60% with lethal hemorrhage being the main reason [15, 16, 19, 26, 35, 42, 44, 46].

In 1989, Beal et al. described THP in a larger patient cohort for the first time. During a three-year period, eight patients sustained a traumatic hemipelvectomy, of whom three survived [1]. Current literature -to our knowledge- reports additional 140 cases, of which a few have been analyzed in larger collectives with sample sizes maximum high as 21 patients [9, 16, 32, 44, 45]. None of these studies reported long-term Quality of Life (QoL) and functional outcomes. Table 1 summarizes the results of studies reporting cohorts > 3 patients. As there is still a lack of information about the functional long-term outcomes and the overall QoL following THP [1, 9, 16, 32, 45].

**Table 1:** Published case series that include more than 3 patients

Author/year	*N*	Sex	Outcome	Age	Side	QoL
Beal et al. [1]	8	N/A	3 survived	N/A	N/A	N/A
Pohlemann et al. [32, 34]	11	11 M	4 survived	21.2	6L,5R	N/A
Wu et al. [45]	4	3F 1 M	4 survived	32	2L,2R	N/A
D’Alleyrand et al. [9]	13	13 M	12 survived	25	6L,7R	N/A
Yu He [16]	21	8 F 13 M	17 survived	31.3	N/A	N/A

*M* male, *F* female, *L* left, *R* right, *N/A* not available

## Materials and methods

A retrospective analysis of all patients with pelvic fractures, that were treated in a level-one trauma center since 1988, was performed. All patients that met the definition of a traumatic hemipelvectomy were selected for evaluation.

The analysis includes demographic data, mechanism of the injury, vital parameters, the Glasgow Coma Scale (GCS) and laboratory parameters at arrival in emergency room, microbiological contamination of the wound and the clinical course.

At a follow-up visit of patients that had survived the long-term functional outcomes, current intake of pain medication and QoL were analyzed.

### Functional outcome at follow-up

To evaluate the functional long-term outcome, follow-up examinations were organized, including standardized scores, as: Timed Up and Go Test (TUG-T), Majeed Score, Merle D’Aubigne Score (MDA Score) and Harris Hip Score (HHS).

The range of motion of the large lower joints was examined using the neutral-zero method. Muscle strength was examined according to Janda [8, 14, 20, 29, 40].

### Quality of Life at follow-up

The health-related quality of life and the psychosocial conditions were analyzed using the 36/12-Item Short-Form Health Survey (SF-36/SF-12) measuring with following eight scales: physical functioning (PF), role physical (RP), bodily pain (BP), general health (GH), vitality (VT), social functioning (SF), role emotional (RE), and mental health (MH) a physical dimension, represented by the Physical Component Summary (PCS), and a mental dimension, represented by the Mental Component Summary (MCS) [5, 24]. The Nottingham Health Profile (NHP) measured six further dimensions, such as energy, sleep, pain, emotional reactions, social isolation, and physical mobility [1, 24]. Data were evaluated according to the instructions given by Bullinger and Kirchberger [5].

### Statistical analysis

Collected data were managed with Microsoft Excel for Mac 2011 (version 14.1.0, Microsoft, Redmond, Washington, US), and, after further processing, analyzed with the statistics program IBM SPSS® Statistics® (version 21, IBM, Armonk, New York, US). However, no statistical analysis was included into this manuscript due to the small patient sample.

## Results

From the pelvic ring fractures treated in a level-A trauma center between 1988 and 2019, 13 patients suffered from THP of whom eight patients survived and five patients died. Detailed medical information of patients are summarized in Table 2. Main reasons for THP were motorcycle accidents (5/13).

**Table 2:** Detailed patient information

Patient	Year of accident	Age	Sex	Side	Outcome	Hip stabilization	GCS	Accident mechanism	Vital parameters (HF + RR)	LA	T(°C)	Hb (mg/dl)	Aptt (sec.)	pH	PBC
1	1988	50	M	R	Died	Hemipelvectomy	3	Overrolling	100 bpm 60/40	N/A	N/A	3.9	N/A	7	60
2	1991	3	M	R	Survived	Hemipelvectomy	N/A	Overrolling	90 bpm 100/75	1.4	37	6.6	52	7.53	20
3	1992	6	M	R	Survived	Hemipelvectomy	14	Overrolling	90 bpm 80/50	N/A	36,6	12	53	7.46	60
4	1997	39	M	R	Died	External fixator + Plate osteosynthesis	N/A	Trapped	110 bpm 80/60	86	36,6	12,8	30,5	7.12	84
5	1997	47	M	L	Died	Plate osteosynthesis	6	Burial	120 bpm 60/40	18.7	36,7	15	N/A	7.23	72
6	1998	35	M	L	Died	External fixator	3	Suicide	120 bpm 85/65	N/A	37	10.6	200	7.34	32
7	2000	25	M	L	Died	Plate osteosynthesis	14	Motorcycle accident	90 bpm 75/35	3.9	37	8.7	N/A	7.3	120
8	1988	38	M	L	Survived	Plate osteosynthesis	3	Motorcycle accident	100 bpm 100/70	N/A	37.1	10.7	51	7.24	115
9	1995	44	M	R	Survived	Plate osteosynthesis	14	Traffic accident	120 bpm 60/35	7.2	37	3.1	N/A	7.3	80
10	2007	18	M	R	Survived	Plate osteosynthesis	15	Motorcycle accident	90 bpm 90/60	0,7	36.5	6,5	49	7.3	50
11	2012	21	M	R	Survived	Hemipelvectomy	15	Motorcycle accident	135 bpm 75/45	3.3	35,9	12,4	30	7.15	64
12	2013	56	M	R	Survived	Hemipelvectomy	5	Explosionstrauma	90 bpm 80/50	6.1	37	5,7	97	7.2	68
13	2013	50	W	R	Survived	External fixator	5	Motorcycle accident	100 bpm 60/40	10.1	37	4,6	128	7.14	79

Vital parameters and blood sample results are the first values at the emergency room

Time the values were all determined from the venous blood by means of blood gas analysis (BGA) in the emergency room

*m* male, *f* female, *R* right, *L* left, *GCS* Glasgow coma scale, *LA* lactat, *T* temperature, *Hb* Hemoglobin, *apt* activated Partial Thromboplastin, *PBC* packed blood cells, *N/A* not available

In five of thirteen patients’ treatment included surgically completion of the traumatic hemipelvectomy resulting in amputation of the affected leg for primary life rescue. Three patients were treated with external fixator; five patients received a plate osteosynthesis. In three cases, control of bleeding was the main reason for amputation. Two patients received a hemipelvectomy because of septical complications.

The most common accompanying injuries were anorectal and urogenital injuries (11/13). Microbiological swabs of the wounds detected gastrointestinal pathogens such as enterococci and enterobacteriacae. Ten patients suffered from septic complications during the following clinical course.

**Fig. 1:**
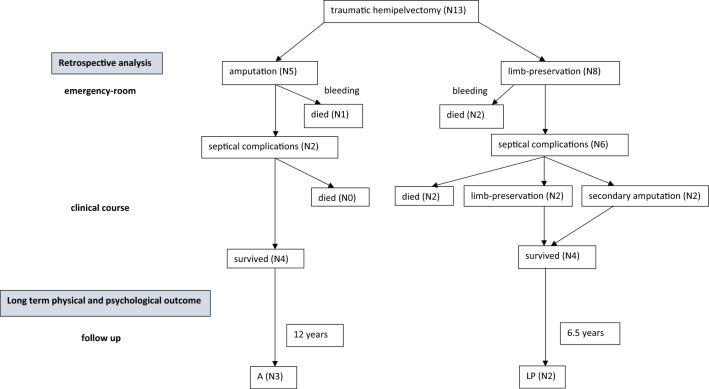
Identified patients suffering from traumatic hemipelvectomy; *A* amputated, *LP* limb-preservation

After primary hemipelvectomy all patients suffered septic complications (Table 3). Due to septic complications, a secondary hemipelvectomy had to be performed on two patients following initial limb salvage, both survived. In the group of the limb-preservation four patients had septic complications, two of them died following septic shock (Fig. 1).

**Table 3:** List of secondary injuries, the pathogenic germ, septical complications and outcome

Patient	Secondary injuries	Pathogen spectrum	Reason for THP	Complications	Outcome
1	Amputation of the testicle, haematothorax	Enterococcus, Enterobacter, Xanthomonas	Bleedingcontroll	Septical	Died
2	Rupture of the bladder and the anal channel	N/A	Bleedingcontroll	Septical	Survived
3	Perineum- rupture, fracture of the femur	N/A	Bleedingcontroll	Septical	Survived
4	Disruption of the ureter and the rectum, fracture of the femur	Enterobacter, Enterococcus		Septical	Died
5	Rupture of the bladder, hämatothorax	N/A		N/A	Died
6	Rupture of the rectum, hämatothorax, fracture of the femur	N/A		Septical	Died
7	Rupture of the rectum, disruption of the ureter	N/A		Septical	Died
8	Disruption of the rectum and bladder	*Bacillus cereus*, *Enterococcus faecalis*		Septical	Survived
9	Fracture of the femur, trauma of the liver vena cava disruption	*E. coli*, *Enterococcus faecalis*, Pseudomonas		Septical	Survived
10	Disruption of the small intestine, rupture of the bladder	*Enterococcus faecalis*, Enterobacter, *E. coli*, *Morganella morganii*		N/A	Survived
11	Disruption of the ureter, psoas-Abscess, disruption of the plexus iliosacralis, laceration of the liver and the kidney	*Enterobacter faecalis*, *Staph aureus*, Achromobacter, *Candida albicans*	Septical complications	Septical	Survived
12	Rupture of vena and arteria iliaca, fracture of the radius	*Enterococcus faecalis*	Septical complications	Septical	Survived
13	Rupture of the bladder, arterial bleeding of the arteria iliaca interna, morel-Lavallée-Lesion	*E. coli*, Enterococcus, *Candida albicans*	Survived	N/A	Survived

*N/A* not available

### Long-term functional outcome 

Five out of 13 patients were available for the follow-up visit on average 11.8 years after the accident. Two patients were treated without amputation (LP) whereas three patient’s emergency surgery included amputation of the affected leg (A). Mean follow-up in the group A was 12 years, in the group LP 6.5 years after traumatic hemipelvectomy.

#### Pelvic scores

**Fig. 2:**
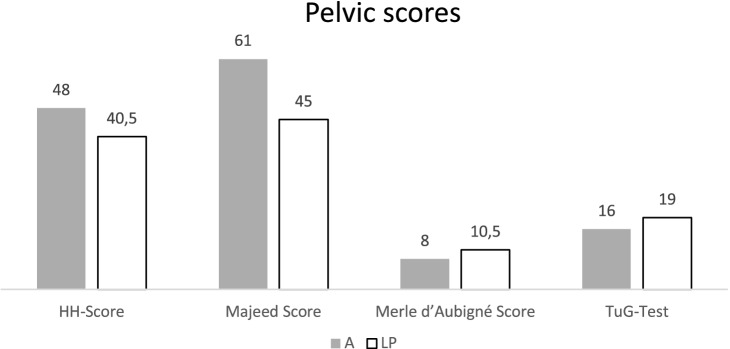
Results of the Pelvic scores; *A* amputated, *LP* limb-preservation

The evaluation of the collected data showed with (A: 48; LP: 40) on average poor results in the Harris Hip Score for both groups [14]. Evaluation of the Merle d'Aubigne score showed a moderate result for group LP (10.5) and a poor result for group A (8) [8]. The Majeed score was unsatisfactory for group LP (45) and satisfactory for group A (60) [29]. The results of the Timed Up and Go Test (TUG-T) are, on average, 16 s (A) and 19 s (LP), which should be rated as a minor mobility restriction without relevance for daily living in both groups [40]. The results of the pelvic scores were balanced in both groups. The Results are shown in Fig. 2.

The daily pain medication intake and their substance class (opioids/non-opioids) were also analyzed. No patient in group A was taking opioids. In group LP, both patients took opioid analgesics daily.

Non-opioid analgesics were taken as concomitant medication in both patients in group LP. In group A, one patient regularly took nonsteroidal anti-inflammatory drugs (NSAID) for pre-existing headaches. Two amputated patients frequently used a wheelchair. One limp preservation and the third amputated patient were able to walk with crutches. One patient after limp preservation needed an ankle foot orthosis (AFO) to walk (Table 4).

**Table 4:** Medical devices

Medical device	Wheelchair	Crutches	Ankle foot orthosis
A (N3)	2	1	
LP (N2)		1	1

*A* amputated, *LP* limb-preservation

### Clinical examination

Muscle strength at the big muscle groups in hip and knee joint and in the upper ankle joint (UAJ) was balanced in both groups, the different values are summarized in Table 5.

**Table 5:** Muscle strength according to Janda of the knee joint, hip joint, upper ankle joint (UAJ), lower ankle joint (LAJ)

Hip joint	Flexion	Extension	Internal rotation	External rotation
A (N3)	5/5	5/5	5/5	5/5
LP (N2)	5/5	4/5	4/5	5/5
*Knee joint*				
A (N3)	5/5	5/5	5/5	5/5
LP(N2)	4.5/5	4.5/5	4.5/5	4.5/5
*LP*^a^	*4.5/5*	*5/5*		

UAJ	Dorsalextension	Plantarflexion	LAJ	Supination	Pronation
A (N3)	3/5	4/5		4/5	3.5/5
LP (N2)	1/5	2/5		3/5	1/5
*LP*^a^	*1/5*	*2/5*		*3/5*	*1/5*

*A* amputated, *LP* limb-preservation

^a^Contralateral side

### Range of motion (neutral-zero-method) of neighbored joints

See Table 6.

**Table 6:** Range of Motion indicated by the neutral zero of the upper ankle joint (UAJ), lower ankle joint (LAJ)

Hip joint (°)	Flexion	Extension	Internal rotation	External rotation
A (N3)	133.3	8.33	35	38.33
LP (N2)	125	5	40	45
*Knee joint (°)*				
A (N3)	120	3.33	16.67	20
LP (N2)	120	5	10	20
LP^a^	120	7.50	5	20

UAJ (°)	Dorsalextension	Plantarflexion	LAJ (°)	Supination	Pronation
A *(N3)*	10	40		40	3.33
LP *(N2)*	0	45		35	0
LP^a^	5	45		35	0

*A* amputated, *LP* limb-preservation

^a^Contralateral side

### Long-term outcome of QoL

**Fig. 3:**
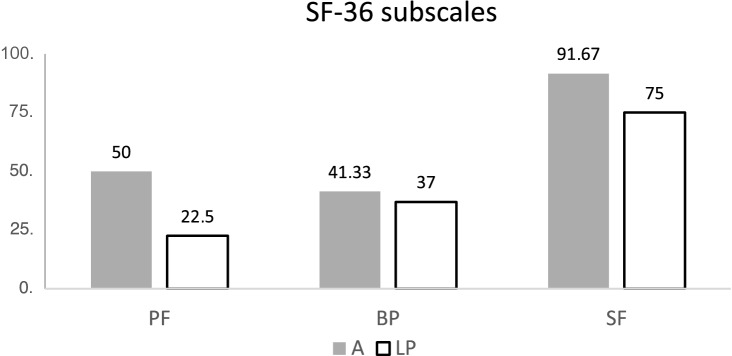
Results of the SF 36 subscales physical functioning (PF), bodily pain (BP) and social functioning (SF); *A* amputated, *LP* limb-preservation

**Fig. 4:**
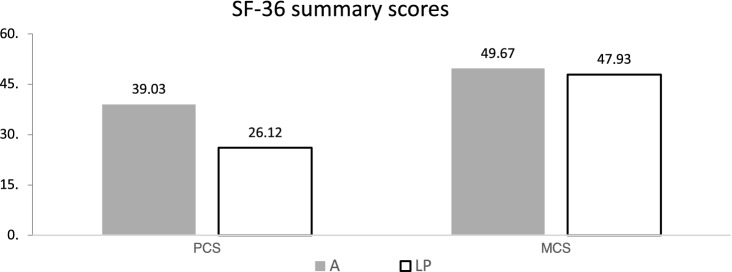
Results of the SF 36 summary scores Mental Component Summary (MCS), Physical Component Summary (PCS); *A* amputated, *LP* limb-preservation

**Fig. 5:**
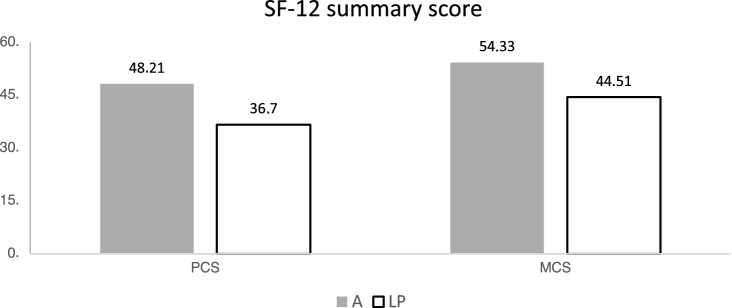
Results of the SF 12 summary scores Mental (MCS) and Physical Component Summary scores (PCS); *A* amputated, *LP* limb-preservation

The analysis of the SF-12/36 demonstrated that the patients who underwent amputation were able to achieve higher values in all recorded sub-areas. Not only did they rate their physical resilience or their social functioning higher, at the same, time they also reported less pain and greater emotional satisfaction. Accordingly, the aggregated total scores (KSK and PSK) of group A were higher than in the LP group (Figs. 3, 4, 5).

**Fig. 6:**
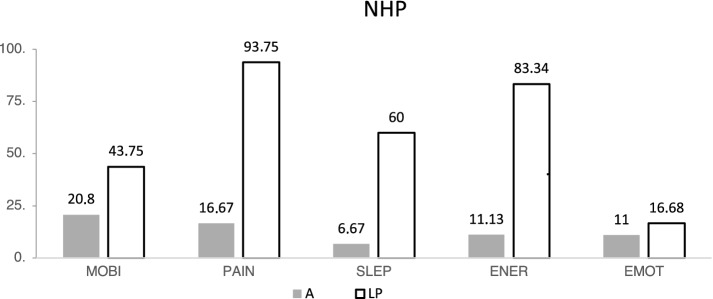
NHP: Results of mobility (MOBI); pain (PAIN); sleep (SLEP); Energy (ENER); Emotion (EMOT) *A* amputated, *LP* limb-preservation

The analysis of the NHP- results revealed differences (Fig. 6), for less pain, a higher sleep quality and general energy level after amputation in the context of a THP.

## Discussion

The existing literature reports the results of case reports and small patient cohorts primarily targeting the survival-rate after traumatic hemipelvectomy, wound healing and related pain following traumatic hemipelvectomy [31, 46]. Here in total, the authors were able to examine long-term outcomes on functional outcome and QoL of five out of 13 patients after traumatic hemipelvectomy and following limb salvage (N2) or amputation (N3).

### Functional outcome

The results of specific hip and pelvic scores were comparable in both groups. The mean Merle d’Aubigne score was better within the LP group. With 10.5 points, the value can nevertheless be assessed as “moderate” [8]. The Majeed Score, Harris Hip Score and the Timed Up and Go Test (TUG-T) results of group A were better than in the LP group [2, 14, 29].

The range of motion and muscle strength measurements for the hip and knee joints and the upper ankle joint revealed no differences in either group. Within the LP and A group abnormal results for ROM of the ankle joint can be related to co-existing peroneal palsy of the contralateral leg (A) and the affected leg (LP) (Table 5) [20, 41]. In the LP group, both patients suffered drop foot due to accident-related peroneal palsy.

Furthermore, the follow-up examination demonstrated that after amputation, the daily mobility seems less restricted according to the results of TUG test [2]. In comparison, the achieved mobility following amputation is satisfactory when comparing these results on functional outcomes to hemipelvectomy outcomes for tumor resection [3, 36]. Even though more nerves and vessels injuries at the pelvis following traumatic hemipelvectomy compared to tumor resections.

Despite daily intake of opioid medication, the reported pain at the pelvic area and the foot could not be controlled sufficiently for the LP subjects. Surprisingly, expecting phantom limb pain, none of the amputees take daily pain medication.

### QoL

Reference groups from the manual by Bullinger and Kirchberger can be used to compare the quality of life of the analyzed two groups to each other and to further musculoskeletal diseases and degenerative diseases as arthritis [5]. In the majority of the analyzed sub-areas of the SF-36 test, the values of the A group were better. The social function (SF) after traumatic hemipelvectomy were higher (91) than patients with musculoskeletal diseases (61) and patients with arthritis (75). In contrast, group LP lags far behind the comparison populations in all categories (75). The group A had less pain (41) compared to the LP (37) and the patients with musculoskeletal diseases (30).

The results for physical function (PF) of group A (50) were superior to the group LP (22) and were similar to the control group of musculoskeletal diseases (51).

Like the SF-36, the results in the SF-12 on both the physical and psychological total scales for group LP are inferior compared to group A and the control groups. The NHP was used and evaluated as an additional screening tool. The Nottingham Health Profile (NHP), together with the SF-36, is one of the standard international instruments for recording quality-of-life [1, 24].

SF-12 results are in concordance with the prior assessed a higher quality of life in the SF-36 for the A group. The individual analysis revealed differences between the two groups in three dimensions. The sleep quality, the general energy level and the level of pain were also better after amputation.

Of clinical interest is the question of post-THP pain [19]. The study found less pain after primary amputation following THP. Despite taking pain medication containing opioids, the LP group reported more frequent and more severe pain than the A group. Pain as long-term sequelae was more common in the group of non-amputee patients, even though a daily intake of opioids. All herein performed statements need to be critically reviewed due to the small cohort. However, in this patient, cohort QoL was better after amputation compared to limb preservation following traumatic hemipelvectomy. Especially the assessment of the patient's subjective QoL will continue to be a very important parameter when assessing the overall outcome [11, 13, 32] and revealed interesting results in this study.

Furthermore, developments in modern prosthetics, socket technologies and assistive devices might allow wider possibilities for those patients regarding pain free mobility and related QoL even in very proximal amputation levels than in the past [18, 37].

The decision on whether to perform a (delayed) primary amputation following THP or not is a very difficult one to make. In contrast to open fractures of the legs there are no scores. But the scores available can possibly help in the decision-making process in traumatic hemipelvectomies. Reconstruction is always the primary aim in trauma surgery, with amputation reserved for exceptional cases as traumatic hemipelvectomy might belong to as nerve and vessels in the pelvis are major injured. In severe open fractures of the leg, there is no difference in outcome between primary and delayed primary amputation, but the prognosis is noticeably worse for secondary than for primary amputations especially regarding development of chronic pain and social consequences related to the high number of revision surgeries during limb salvage and chronification of pain [12].

Once a traumatic hemipelvectomy has been diagnosed, surgical completion could ensure the survival of patients and could presumably cause fewer septic complications. Long-term quality of life after amputation also appears to be good [22, 25, 27, 35, 38]. In conclusion to this study, the authors share this approach to traumatic hemipelvectomies and rely on the presented results pointing out a tendency to a superior functional and mental situation after amputation compared to limb preservation. However, as to the small patient cohort only, deriving any recommendations for treatment of course is difficult. A multicentric study or a metaanalytical view of the work of recent decades could improve validity, power and thus the basis for decision-making.
